# Misophonia is related to stress but not directly with traumatic stress

**DOI:** 10.1371/journal.pone.0296218

**Published:** 2024-02-22

**Authors:** Rachel E. Guetta, Marta Siepsiak, Yanyan Shan, Emily Frazer-Abel, M. Zachary Rosenthal

**Affiliations:** 1 Center for Misophonia and Emotion Regulation, Department of Psychiatry and Behavioral Sciences, Duke University Medical Center, Durham, North Carolina, United States of America; 2 Department of Psychology and Neuroscience, Duke University, Durham, North Carolina, United States of America; 3 Department of Psychology, University of Warsaw, Warsaw, Poland; Qatar University College of Nursing, QATAR

## Abstract

The relationship between misophonia, stress, and traumatic stress has not been well characterized scientifically. This study aimed to explore the relationships among misophonia, stress, lifetime traumatic events, and traumatic stress. A community sample of adults with self-reported misophonia (*N* = 143) completed structured diagnostic interviews and psychometrically validated self-report measures. Significant positive correlations were observed among perceived stress, traumatic stress, and misophonia severity. However, multivariate analyses revealed that perceived stress significantly predicted misophonia severity, over and above traumatic stress symptoms. The number of adverse life events was not associated with misophonia severity. Among symptom clusters of post-traumatic stress disorder, only hyperarousal was associated with misophonia severity. These findings suggest that transdiagnostic processes related to stress, such as perceived stress and hyperarousal, may be important phenotypic features and possible treatment targets for adults with misophonia.

## Introduction

Misophonia is a sound intolerance disorder marked by strong aversion and heightened multi-modal (i.e., physiological, cognitive, behavioral) emotional reactivity to certain repetitive auditory stimuli usually produced by other humans (e.g., oral or facial cues such as chewing, sniffing, throat clearing) [1, 2]. First described by Jastreboff and Jastreboff in 2001 [3], misophonia is a newly studied phenomenon that is gaining empirical and clinical attention across health disciplines. Emotional responses in anticipation or response to aversive cues can include anxiety, anger and disgust, increased sympathetic nervous system activation (e.g., heart rate, muscle tension), and behavioral urges and actions that function as freeze (e.g., hypervigilance), flight (e.g., escape or avoidance), and fight (e.g., confrontation, verbal aggression, indirect aggression; rarely physical aggression) reactions. These patterns collectively contribute to chronic and significant psychological distress and impairment in social, academic, or occupational functioning. Contextual factors (e.g., certain social settings, particular people, affective state) may influence the severity of misophonic responses [2]. Early studies using self-report questionnaires pointed to the possibility that misophonia co-occurs with obsessive compulsive disorder [4] and other specific psychiatric disorders. However, the most recent research using structured diagnostic interviews has observed different results, indicating that (a) there do not appear to be any specific psychiatric disorders uniquely associated with misophonia and (b) the most commonly associated psychiatric disorders, when assessed carefully and comprehensively, may be current anxiety disorders, features of obsessive compulsive personality disorder, and a lifetime history of mood disorders [5–7].

One approach to investigate the relationship between misophonia and other conditions is to examine transdiagnostic mechanisms that may either be shared or can explain differences between disorders. Mechanisms of stress, defined broadly, are of particular interest, given the prevalence of stress reactions in misophonia across affective over-reactivity (e.g., anger, aggression), flight or fight behaviors, physiological arousal (e.g., activation of the sympathetic nervous system), attentional difficulties (e.g., hypervigilance to triggers), and overall distress [2].

There is a growing body of evidence indicating that stress may be a causative factor in the development of sound over-responsivity. Mazurek et al. demonstrated that behaviorally induced stress, including acoustic stress, can result in transient auditory sensitivity in rats, which may be viewed as an adaptive process of hypervigilance in an insecure environment [8]. Recently, Manohar et al. showed that chronic stress induced pharmacologically with corticosterone hormones caused an increase in sound over-responsivity and auditory cortex hyperactivity in rats [9]. Similar effects of stress on sound perception have also been observed in humans. For instance, in an experimental study, Hasson et al. found that sound sensitivity reflected in uncomfortable loudness levels (ULL) significantly increased in a group of women after stress exposure [10]. Emotional exhaustion was also negatively correlated with ULL and hyperacusis in non-clinical participants [11]. Nevertheless, the relationships between misophonia and stress has not yet been carefully examined.

Stress, however, is a complex and multi-faceted construct that implicates biological and psychological systems, necessitating specificity in measurement to glean empirically and clinically useful findings. At the broadest level, reactions to everyday stressors manifest in physiological and biological changes (e.g., increases in heart rate, galvanic skin response, activation of the sympathetic nervous system), though the impact of reactions are mediated by subjective appraisals of the stressors [12]. Perceptions of stress can vary greatly based on individual differences in factors ranging from information processing to cognitive style, and attentional biases to coping strategies [13, 14]. Perceived stress, or the degree to which individuals appraise their lives and everyday situations as unpredictable, uncontrollable, or overloading [15] is a transdiagnostic factor associated with risk for development of psychiatric, behavioral, and physical health problems [16–18], highlighting the importance of subjective appraisals in understanding short- and long-term consequences of situational and chronic stress. Decades of research have evidenced distinct biological pathways that mediate the relationships between traumatic events and perceived stress on various health outcome measures [15, 17]. The differences in explanatory and mechanistic pathways between perceived stress, presence of stressful life events, and adverse health outcomes further highlights the need for specificity in measuring stress as a multidimensional construct.

In addition to probing perceived stress, it is important to measure history of traumatic life events. To move towards an objective consensus definition of trauma, the stressor criterion (Criterion A) used to assess and diagnose posttraumatic stress disorder (PTSD) includes specific categories of events that one can experience directly, witness, or learn about via repeated and extreme exposure [19]. Endorsement of these events can be catalogued in self-report measures, such as the Life Events Checklist for *DSM-5* (LEC-5) [20]. Still, the development of PTSD is broadly conceptualized as an interruption to natural recovery from a trauma, as the majority of individuals will not meet criteria for a PTSD diagnosis three months following exposure to a Criterion A event [21]. Stated differently, epidemiological work indicates that most adults have experienced at least one potentially traumatizing event across their lifetimes, but under 10% develop PTSD [21, 22]. As such, traumatic stress, including PTSD and its various symptom clusters, must be measured in addition to the pure presence and frequency of traumatic life events. In addition to presence of a Criterion A event, PTSD symptoms fall into four clusters: re-experiencing (e.g., intrusive thoughts, flashbacks, nightmares), avoidance (e.g., suppression of trauma-related thoughts, avoidance places or people that are reminiscent of the trauma) negative cognitions and mood (e.g., persistent negative emotions, increased guilt and shame, negative belief about self and others), and hyperarousal (e.g., hypervigilance, startle reaction). Some of these symptoms may not be distinct to PTSD, and may reflect transdiagnostic factors unrelated to trauma specifically (e.g., avoidance, negative alterations in mood and cognition).

Lastly, large scale stressors affecting individuals at a population level are an additional dimension of stress that is important to capture empirically. Studies implementing self-report measures of stress and traumatic stress may at times be confounded by cohort-level current or state stress. The COVID-19 pandemic is a good example of this, wherein self-reported stress may at times be confounded by state level stress that is ongoing for individuals. As such, it is important to not ignore population-level stressors that may contribute to or account for self-reported stress.

The question of if and how misophonia may be related to these various components of stress, however, has been largely unaddressed. Most studies to date assessing the relationships between misophonia and stress have focused on the prevalence of traumatic stress, particularly the comorbidity with participant self-reported diagnosis [23] of PTSD or diagnostic conclusions from structured interviews [6, 7]. Among these studies, there have been mixed findings on the extent of the relationship between PTSD and misophonia.

On the one hand, some work suggests that misophonia may be related to PTSD [23]. In an online study among a sample of 301 adults with self-reported misophonia, Rouw and Erfanian found that PTSD was the most frequently self-reported co-occurring diagnosis (12% of sample) of 10 conditions probed with self-report items (PTSD, OCPD, tinnitus, hyperacusis, auditory processing disorder, attention deficit disorder, exploding head syndrome, phonophobia, eating disorders, and selective mutism) [23]. Findings from that study indicate that only PTSD was significantly related to misophonia severity. Of note, however, this study relied solely on self-reported assessments of 10 idiosyncratically chosen psychiatric and medical co-occurring disorders and did not use as comprehensive of an assessment approach as more recent studies [5–7]. In the absence of more rigorous diagnostic approaches, it is not possible to draw definitive conclusions about the relationship between misophonia and PTSD.

With that caveat in mind, several recent studies have found relatively low rates of PTSD in misophonia when using structured psychometrically valid assessment measures to explore the relationships among misophonia and associated mental health conditions [6, 7, 24]. In one study in Amsterdam, 1.7% of adults (*N* = 575) seeking treatment for misophonia met criteria for PTSD when assessed with the Mini International Neuropsychiatric Interview (M.I.N.I.; Sheehan et al., 1998 [25]), which assesses 15 current psychiatric problems [6]. In another study in Poland, Siepsiak et al. used the M.I.N.I. and found that 11.9% of adults with misophonia met criteria for PTSD [25]. Rosenthal et al. conducted the first study to characterize *DSM-5* [7] disorders using the Structured Clinical Interview for *DSM-5* [26] in a sample of 207 adults in the U.S. with high misophonia symptoms. Results indicated that 2.9% had concurrent PTSD, with 15.5% having PTSD at some point in their lifetime [7]. Based on the findings in these three carefully conducted studies, misophonia appears to be associated with relatively low or modest rates of PTSD at a prevalence somewhat congruent with what may be observed in the general population (between 6.8–10%) [21, 22].

Accordingly, from these recent studies one could conclude that people with misophonia may not be particularly likely to be diagnosed with current PTSD, raising doubt about whether misophonia should be conceptually related to a history of traumatic stress. However, we believe that conclusion is premature, and that more research is needed to help better understand whether and how misophonia may be related to traumatic stress. No studies have examined whether misophonia is associated with stressful events across the lifespan. It is possible that PTSD itself may not be related to misophonia, but rather that cumulative adverse life events may, as is the case with many physical and mental health problems, be associated with the etiology and/or course of misophonia [27, 28].

A diagnosis of current PTSD does not appear to be directly linked with misophonia, but what about the broader construct of stress? One recent study investigated the relationship between misophonia symptoms and dimensional aspects of traumatic stress [29]. These findings suggested a moderate positive correlation between misophonia symptoms and arousal and intrusions, and a low correlation with avoidance. However, it should be noted that this study was conducted on a sample of hospitalized patients with depression; additional research using a similar approach is needed with outpatient and community samples before clear conclusions can be made.

No studies have explored the perceptions of stress in relation to misophonia, or broader population-level stressors. Certainly, some clusters of PTSD symptoms (e.g., avoidance, negative alterations in mood and cognition) are also transdiagnostic factors, unrelated to trauma specifically, but associated with other mental health problems (e.g., mood disorders). It is unknown to what extent some of these shared processes are also related to misophonia outside of the context of trauma. For example, hyperarousal is central to the clinical picture of misophonia, both in heightened physiological reactions to trigger sounds (e.g., increased heart rate, sweating), and in hypervigilance towards trigger sounds and related contextual cues. Avoidance of potentially triggering situations and individuals is also central to maintenance and impairment of misophonia. As such, investigating how specific components of PTSD overlap or diverge from misophonia, over and above PTSD diagnosis, is warranted. One possible explanatory model may be that certain symptom clusters of PTSD could be related to misophonia severity.

Another possibility is that perceived stress more broadly, that does not result in chronic traumatic stress, may be related to misophonia severity. Firstly, given the empirical evidence that perceived stress is transdiagnostic and associated with greater psychological distress, and medical and psychiatric problems broadly [16–18], it makes conceptual sense that subjective appraisals of stress would be related to misophonia severity as well. Further, the anecdotal experiences of misophonia often involves a profound sense of unpredictability, uncontrollability, and inescapability [2], suggesting the importance of subjective appraisal of stress reaction in misophonia.

Understanding the ways in which cumulative adverse life events, PTSD diagnosis, PTSD symptomatology, and perceptions of stress are related to misophonia may help shed light on the etiology and maintenance of misophonia, as well as inform treatment recommendations. The primary aim of the current is to better understand the relationships among misophonia, stress, and trauma in a community sample. A secondary aim is to preliminarily examine mechanisms of trauma and stress-related sequalae (i.e., clusters of PTSD symptoms via the PCL-5) that contribute to misophonia severity.

We hope to deepen an understanding of whether traumatic life experiences are associated with misophonia, as well as how perceptions of stress interact with misophonia severity. Because this study was conducted during the peak of COVID-19, we also measured stress in the context of the pandemic in order to account for this population-level stressor and to reduce the possibility confounding results of stress related to COVID-19. Based on the relatively low prevalence of PTSD in prior studies [6, 7, 24] the theoretical and empirical associations between mechanisms of stress and psychological distress broadly, we hypothesized that specific components of stress (e.g., perceived stress, certain clusters of PTSD) rather than PTSD diagnosis itself would be more strongly related to misophonia severity.

## Materials and methods

### Participants and study design

A sample of 143 adults (average age = 36.89 years; females = 67.8%; see Table 1 for demographic information) residing in the U.S. and who identified as having auditory sound sensitivity enrolled in the current study via a REDCap link posted to the Duke Center for Misophonia and Emotion Regulation website [30]. The Duke Health Institutional Review Board (IRB) approved study procedures and all participants provided written informed consent before participation. Study visits were conducted between December 9, 2019 and December 2, 2022, and included administration of the SCID-5 by trained assessors, followed by several self-report questionnaires that participants were asked to fill out on their own (see below).Exclusion criteria for enrollment included current psychotic disorder, current mania, and current anorexia nervosa; eligibility was confirmed via a phone screening using the M.I.N.I. following completion of the REDCap online screening form. Participants received $75 USD for completion of structured clinical interviews and self-report assessments.

**Table 1:** Demographic characteristics of the current sample.

Characteristic	*n*	%
Age in years (*M*, *SD*)	36.88	12.84
**Sex**		
Male	43	30.1
Female	100	69.9
**Gender Identity**		
Male	43	30.1
Female	97	67.8
Genderqueer	1	.7
Other	2	1.4
**Sexuality**		
Straight	109	76.2
Gay	8	5.6
Bisexual	14	9.8
Something else	6	4.2
Don’t know	5	3.5
Did not disclose	1	.7
**Race**		
White	106	74.1
African American	6	4.2
Native American	2	1.4
Chinese or Chinese American	7	4.9
Other Asian	6	4.2
Other	3	2.1
More than one race	13	9.1
**Hispanic/Latinx**		
Yes	23	16.1
No	120	83.9
**Income Level**		
0-$10,000	21	14.7
10,001 - $65,000	39	27.27
65,001 –more than $100,000	83	58.04
**Marital Status**		
Single	58	40.6
Married	61	42.7
Separated	4	2.8
Divorced	5	3.5
Living with partner	14	9.8
Missing	1	.7

*N* = 143

### Measures

#### Misophonia Questionnaire (MQ) [31]

The MQ is a three-part self-report questionnaire that consists of: (a) presence of misophonic triggers (subscale 1), (b) emotional and behavioral responses to misophonic triggers (subscale 2), and (c) a single-item self-rated impairment of sound sensitivities. Participants completed the MQ as part of the online screen posted on our Center’s website before enrolling in the study visit. Self-rated MQ impairment ranged from 1 to 12, indicating a sample with misophonia impairment across the spectrum from mild to severe; average self-rated impairment was 7.53 (*SD* = 2.03) corresponding with “moderate sound sensitivities.” Cronbach’s alpha in the current sample was *α* = .84 (McDonald’s omega = .83).

#### Structured Clinical Interview for Diagnostic and Statistical Manual-5^th^ research edition (SCID-5) [32]

The SCID-5 is a widely used, validated semi-structured interview designed to assess diagnostic symptoms of *DSM-5* disorders. Trained assessors included licensed clinical psychologists, as well as clinical psychology postdoctoral fellows and doctoral students. Interviewers assessed for categorical diagnoses of current (e.g., past month, past six months) and lifetime disorders. Composite variables were also created to capture whether participants met criteria for current or lifetime categories of disorders (i.e., any mood, anxiety, substance use, or trauma-related disorder). Diagnostic variables were coded as either 0 (below threshold of meeting clinical criteria) or 1 (meeting full criteria for the presence of disorder). Validation studies for the SCID-5 have evidenced strong internal consistency (*αs* > .80) [33]. Inter-rater reliability for the SCID-5 interviews was assessed by a blind rater randomly rating 8% of recorded interviews. Significant Cohen’s κ ranged from 0.63 to 1.00 (*ps* < 0.05) for all trauma- and stressor-related disorders.

#### Stressful Life Events Checklist for *DSM-5* (LEC-5) [20]

The Life Events Checklist for the *DSM-5* (LEC-5) is a self-report measure designed to screen for potentially traumatic events over an individual’s lifetime. The LEC-5 is often used to establish exposure to a PTSD Criterion A traumatic event. This self-report tool assesses for the 16 events known to potentially result in PTSD (e.g., natural disaster, physical or sexual assault, combat).

#### Posttraumatic Stress Disorder Checklist for *DSM-5* (PCL-5) [34]

The PCL-5 is a 20-item, psychometrically validated self-report measure that assesses symptoms of PTSD in accordance with the *DSM-5* diagnostic criteria. The PCL-5 is often used to screen for the possible presence of PTSD and/or severity of PTSD symptoms. Respondents are asked to rate each item in terms of how much they were bothered by that symptom. Each item is captured on a Likert scale ranging from 0 (“not at all”) to 4 (“extremely”). A total symptom severity score ranges from 0–80, calculated by summing each item’s 0–4 response. Preliminary research suggests PCL-5 score between 31–33 indicates probable PTSD across samples [35]. Validation studies reflect strong internal consistency (*α* = .95) [34]. Cronbach’s alpha in the current sample was *α* = .93 (McDonald’s omega = .93).

#### Perceived Stress Scale (PSS) [15]

The PSS is a widely used self-report tool that measures perceptions of stress in the past month. The 10 items probe frequency of perceived stress and associated experiences (e.g., confidence in handling the stressful situation, ability to overcome stressful situations). Each item is captured on a 5-point Likert scale, ranging from 0 (“never”) to 4 (“very often”). Total scores on the PSS range from 0–40 with higher scores indicating greater levels of perceived stress. Scores ranging from 0–13 are considered low perceived stress, scores between 14–26 are considered moderate perceived stress, and scores 27–40 are considered high levels of perceived stress. Validation studies reflect strong internal consistency (*α* = .91) [36]. Cronbach’s alpha in the current sample was *α* = .89 (McDonald’s omega = .90).

#### Acute Stress Disorder Scale—Adapted for COVID

The Acute Stress Disorder Scale (ASDS-C) was designed to assess the psychological impact of the COVID-19 pandemic. This adapted version for COVID-19 was adapted from the Acute Stress Disorder Scale (ASDS) [37], a self-report measure of acute stress disorder symptoms following a traumatic event. The ASDS-C frames all stress-related questions as they relate to COVID-19 (i.e., “Have you tried not to think about [COVID-19]?”). The ASDS-C consists of 20 total items, each rated a 5-point scale from 1 (“not at all”) to 5 (“very much”). Total scores on the ASDS-C can range from 20–100; total scores above a 56 indicate significant reactions to COVID-19. Although the adapted ASDS-C has not been psychometrically validated, the original ASDS has strong internal consistency (*α* = .96) [37]. Cronbach’s alpha in the current sample was *α* = .94 (McDonald’s omega = .94).

#### Duke Misophonia Questionnaire (DMQ) [38]

The Duke Misophonia Questionnaire (DMQ) is a psychometrically validated self-report measure of misophonia using factor analytic procedures combined with IRT in an English-speaking sample. The DMQ has 86 items and includes subscales: 1) trigger frequency (16 items), affective responses (8 items), 3) physiological responses (5 items), 4) cognitive responses (10 items), 5) coping Before (6 items), 6) Coping During (10 items), 7) Coping After (5 items), 8) Impairment (12 items), and Beliefs (14 items). Composite scales are derived from overall Symptom Severity (combined Affective, Physiological, and Cognitive Subscales) with scores ranging from 0–83 and Coping (which combined the three coping subscales: before, during, and after being triggered), with scores ranging from 0–78. Clinical impairment scores, derived from the Impairment Subscale, ranging from 0–13 are considered "minimal-mild impairment,” scores between 14–38 are considered "moderate impairment," and scores between 39–48 are considered "severe to very severe impairment.” Internal consistency results indicated that subscale intercorrelations were all within the range of .43–0.84, evidencing strong relationships between the proposed constructs. The mean clinical impairment score in the current sample was 13.43 (*SD* = 10.18), indicating that on average impairment in this sample is on the high end of mild impairment. In frequency of trigger sounds, 39.9% endorsed being triggered between 2–5 times per day on average. The mean symptom severity composite score, combining affective, physiological, and cognitive subscales, was 43.76 (*SD* = 17.73). The mean coping composite score was 35.72 (*SD* = 14.04).

### Data analytic plan

All analyses were conducted in SPSS 27.0 [39] and JASP 17.1 [40]. First, we explored the frequency of current and lifetime PTSD diagnoses via the SCID-5 in the current sample, as well as the frequency of potentially traumatic events (LEC-5). Bivariate correlations were conducted to examine if frequency of stressful events across the lifespan (via the LEC-5) was associated with misophonia. Next, Pearson’s bivariate correlations were conducted to examine the relationships among misophonia symptoms (via the DMQ) and (a) PTSD symptoms, including clusters of reexperiencing, avoidance, negative alterations in mood and cognitions, and hyperarousal (PCL-5), (b) acute stress disorder scale, adapted for COVID-19 (ASDS-C), and (c) perceived stress (PSS total score). In order to examine misophonia severity most comprehensively, we created a composite DMQ severity score by summing the standardized z-scores of the DMQ symptom subscale and the DMQ impairment subscale.

We then conducted multiple linear regressions in order to explore if (a) diagnosed trauma-related disorders and (b) particular components of stress and trauma (PCL-5, PSS) predict misophonia severity (DMQ), controlling for age, sex, and frequency of traumatic events across the lifespan.

Last, in order to visualize the partial correlation network among our variables of interest, we conducted network analysis in JASP which makes use of the R package qgraph [41]. We applied a graphical least absolute shrinkage and selection operator (gLASSO) [42] regularization, which sets small or unstable correlations within the network to zero to better interpret a parsimonious network. The Extended Bayesian Information Criterion (EBIC) [43] was applied to select the optimal network model derived from the gLASSO solutions. The tuning parameter was set to .5 for increased parsimony and interpretability (i.e., higher sensitivity and specificity, and fewer edges). The accuracy of edge weights was assessed by calculating 95% confidence intervals based on non-parametric bootstrapping (*n* = 1,000 boots), in line with the recommendation for LASSO regularized edges [41].

## Results

### Descriptive statistics

In the current sample, 32.9% (*n* = 47) of participants met criteria for at least one lifetime trauma and stressor-related disorder, including PTSD, ASD, adjustment disorder, or another specified trauma- and stressor-related disorder. As shown in Table 2, 11.9% (*n* = 17) met current criteria for any trauma-related disorder (PTSD, ASD, adjustment disorder, other specified trauma- and stressor-related disorder). Among current diagnoses of trauma and stressor related disorders, only 3.5% met full criteria for PTSD, whereas Other specified trauma disorder was most commonly diagnosed.

**Table 2:** Frequency of SCID-5 trauma- and stressor-related disorders.

		Frequency (*N =* 143)	Percentage
**Current**			
	PTSD	5	3.5%
	ASD	0	0%
	Adjustment disorder	4	2.8%
	Other specified trauma disorder	8	5.6%
	Any trauma disorder	17	11.9%
**Lifetime**			
	PTSD	31	21.7%
	Other specified trauma disorder	17	11.9%
	Any trauma disorder	47	32.9%

**Note**: PTSD = Posttraumatic Stress Disorder; ASD = Acute Stress Disorder

Descriptive results from the LEC-5 reflect that the most frequently reported potentially traumatic events experienced directly were transportation accidents (51.7%), unwanted or uncomfortable sexual experiences (36.4%), natural disasters (32.2%), sexual assault (21.7%), and sudden and unexpected death of a loved one (29.4%). Additionally, 30.1% of participants self-reported experiencing other highly stressful events. See Table 3 for more details. Bivariate correlations evidenced that frequency of lifetime stressful events was not associated with misophonia across DMQ symptoms score (*r* = .02, *p* = .799), DMQ impairment score (*r* = .09, *p* = .325), and the composite DMQ severity score (*r* = .06, *p* = .499).

**Table 3:** Prevalence of traumatic events experienced via the Life Events Checklist for *DSM-5*.

LEC-5 event	Frequency (*N =* 143)	Percentage
Transportation accident	74	51.7
Other unwanted sexual experience	52	36.4
Natural disaster	46	32.2
Other very stressful event	43	30.1
Sudden, unexpected death of loved one	42	29.4
Physical assault	31	21.7
Sexual assault	31	21.7
Work or recreation accident	16	11.2
Assault with a weapon	14	9.8
Life-threatening illness or injury	13	9.1
Fire or explosion	9	6.3
Exposure to toxic substance	7	4.9
Severe human suffering	3	2.1
Sudden, violent death	3	2.1
Combat or exposure to warzone	1	.7
Captivity	1	.7
Personally causing serious injury, harm, or death	1	.7

Note: LEC-5 = Life Events Checklist for *DSM-5*.

### Correlations between misophonia and variables of stress and trauma

In order to explore the relationships between measures of stress and trauma with misophonia, we conducted bivariate correlations among DMQ symptoms score, DMQ impairment score, and the composite DMQ severity score measures of current PTSD symptoms (PCL-5 total and subscale scores), perceived stress (PSS), and COVID-related stress (ASDS-C); see Table 4. DMQ symptoms, impairment, and severity were significantly correlated with PCL-5 total score and several subscales, particularly the hyperarousal subscale. DMQ scores were also significantly correlated with perceived stress and COVID-related stress.

**Table 4:** Bivariate correlations among misophonia severity and variables of trauma and stress.

	PCL-5 total	PCL-5 reexperiencing	PCL-5 avoidance	PCL-5 Negative alterations	PCL-5 hyperarousal	PSS total	ASDS-C total
DMQ symptoms	.194*	.136	.102	.146	.262**	.322**	.226**
DMQ impairment	.293**	.204*	.122	.252**	.364**	.363**	.258**
DMQ severity	.266**	.186*	.123	.218*	.342**	.374**	.264**

**Note**: DMQ = Duke Misophonia Questionnaire; PCL-5 = Posttraumatic Stress Disorder Checklist for *DSM-5*; PSS = Perceived Stress Scale; ASDS-C = Acute Stress Disorder Scale–Adapted for COVID-19.

** = *p* < .001;

* = *p* < .05

### Relationship among misophonia and components of stress and trauma

First, we conducted a multiple linear regression to examine if either a current or lifetime diagnosis of a trauma-related disorder via the SCID-5 predicted misophonia severity (DMQ severity composite). Neither a current nor lifetime diagnosis of a trauma related disorder predicted misophonia severity (F(4, 138) = 1.57, *p* = .186). Models with DMQ symptom score and DMQ impairment score entered as the dependent variables were also not significant.

Next, we examined the relationship among misophonia severity and variables of stress and trauma with a multiple linear regression (Table 5). As has been done in previous studies using similar analyses to characterize misophonia [44], Step 1 controlled for age and sex. Step 2 included current PTSD symptoms (PCL-5), COVID-related acute stress (ASDS-C), and perceived stress (PSS). This model accounted for a significant proportion of the variance in overall misophonia severity (F(5, 129) = 4.56, *p* < .001, *R*^*2*^ = .15, *R*^*2*^ change = .13). However, only PSS (standardized β = .30, *t*(2.97), *p* = .044) total score predicted misophonia severity.

**Table 5:** Multiple linear regression examining variables of stress on trauma on misophonia severity.

Dependent Variable		Variables	SE	β std	p	R^2^	R^2^ change
DMQ Severity	Step 1	Age	.01	.02	.803		
		Sex	.34	.14	.107		
	Step 2	Age	.01	.06	.451	.15	.13
		Sex	.33	.09	.281		
		PCL-5	.02	.02	.863		
		ASDS-C	.01	.10	.349		
		**PSS**	**.03**	**.30**	**.004**		

**Note**: DMQ = Duke Misophonia Questionnaire; PCL-5 = Posttraumatic Stress Disorder Checklist for *DSM-5*; ASDS-C = Acute Stress Disorder Scale–Adapted for COVID-19; PSS = Perceived Stress Scale.

In order to examine if number of stressful life events impact the relationship between perceived stress and misophonia severity, we added total events endorsed via the LEC-5 as a covariate in Step 1 of another model (Table 6). Perceived stress remained a significant predictor of misophonia severity, over and above the presence and/or frequency of traumatic stress (F(6,128) = 3.78, *p* = .002, *R*^*2*^ = .15, *R*^*2*^ change = .12).

**Table 6:** Multiple Linear Regression Accounting for number of stressful life events on misophonia severity.

Dependent Variable		Variables	SE	β std	p	R^2^	R^2^ change
DMQ Severity	Step 1	Age	.13	-.01	.942		
		Sex	.35	.15	.086		
		LEC	.01	.10	.292		
	Step 2	Age	.01	.07	.425	.15	.12
		Sex	.34	.09	.315		
		LEC-5	.02	-.03	.785		
		PCL-5	.02	.03	.823		
		ASDS-C	.01	.10	.335		
		**PSS**	**.03**	**.30**	**.004**		

**Note**: DMQ = Duke Misophonia Questionnaire; LEC-5 = Life Events Checklist; PCL-5 = Posttraumatic Stress Disorder Checklist for *DSM-5*; ASDS-C = Acute Stress Disorder Scale–Adapted for COVID-19; PSS = Perceived Stress Scale.

### Network analysis to visualize relationships among misophonia, stress, and trauma

**Fig 1:**
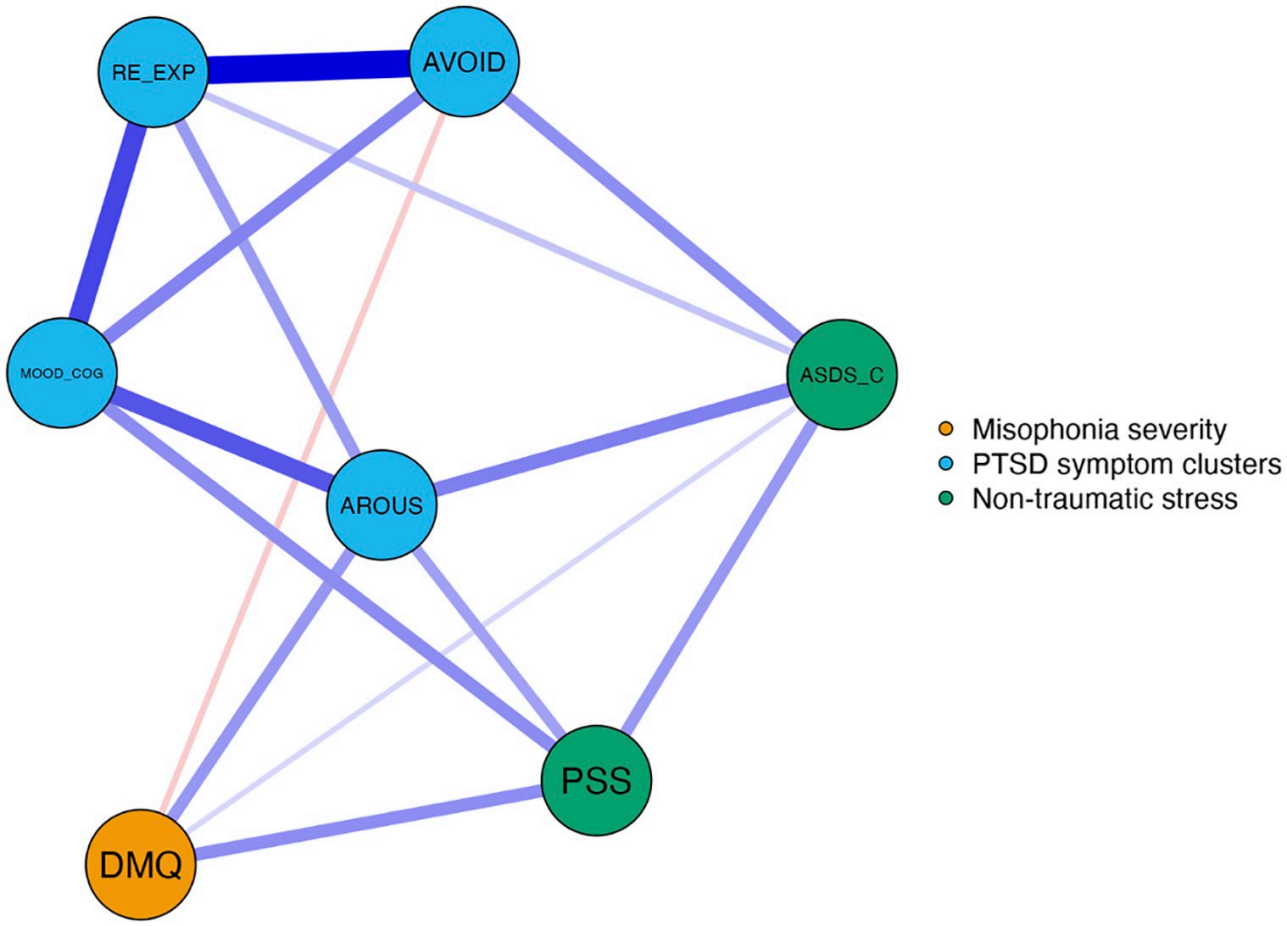
Network plot of variables of misophonia, stress, and trauma. *Note*: Estimated EBIC gLASSO network of misophonia severity, perceived stress, clusters of PTSD symptoms, and stress during COVID-19 pandemic. DMQ = Duke Misophonia Questionnaire; PSS = Perceived Stress Scale; ASDS-C = Acute Stress Disorder Scale Adapted for COVID-19; MOOD_COG = Alterations in mood and cognitions; RE-EXP = Re-experiencing; AVOID = Avoidance; AROUS = Hyperarousal.

The EBICGlasso network including misophonia severity (DMQ severity composite score), PTSD symptoms (PCL-5 re-experiencing, avoidance, negative alterations in mood and cognitions, and hyperarousal clusters), perceived stress (PSS), and stress during COVID-19 (ASDS-C), is displayed in Fig 1. There were seven nodes and 15 non-zero edges in the network. Thicker links (‘edges’) represent stronger correlations and thinner edges reflect weaker correlations; blue lines represent positive correlations, and orange lines reflect negative correlations. Nodes with stronger connections are placed closer together than nodes with weaker connections. Perceived stress and hyperarousal both had positive associations with misophonia severity (*r*s = .19, .18, respectively). Acute stress in the context of COVID-19 had a weak association with misophonia severity (*r* = .07) and avoidance had both a weak and negative association with misophonia severity (*r* = -0.08).

Last, to further our secondary aim of exploratory analyses to understand how symptoms of stress and trauma relate to symptoms of misophonia, we conducted a forced entry regression to examine which cluster(s) of PTSD via the PCL-5 most account for misophonia severity (DMQ severity; see Table 7). Because earlier analyses indicated that perceived stress predicted misophonia severity over and above PTSD symptoms, we included PSS total score as a covariate in Step 1 of the model. Of the four subscales representing the four clusters of PTSD symptoms (reexperiencing, avoidance, negative alterations in mood and cognition, and hyperarousal), only hyperarousal significantly accounted for variance in misophonia severity (F(7,128) = 4.20, *p* < .001, *R*^*2*^ = .19, *R*^*2*^ change = .04).

**Table 7:** Regression examining contribution of current PTSD symptom clusters on misophonia severity.

Dependent Variable		Variables	SE	β std	p	R^2^	R^2^ change
DMQ Severity	Step 1	Age	.01	.07	.379		
		Sex	.33	.13	.1086		
		**Perceived stress**	.02	.35	**< .001**		
	Step 2	Age	.01	.05	.569	.19	.04
		Sex	.33	.13	.111		
		**Perceived stress**	**.03**	**.27**	**.007**		
		Re-experiencing	.07	.02	.888		
		Avoidance	.12	-.10	.399		
		Negative alterations	.04	-.07	.576		
		**Hyperarousal**	**.05**	**.27**	**.022**		

**Note**: DMQ = Duke Misophonia Questionnaire.

## Discussion

The primary aim of this study was to better understand the relationships among misophonia, stress, and traumatic stress in a community sample of U.S. adults. We examined (a) the prevalence of trauma-related disorders using structured diagnostic interviews (e.g., PTSD, ASD, adjustment disorder) and history of stressful life events, (b) the impact of trauma-related diagnoses and stressful life event histories on misophonia, and (c) whether traumatic stress specifically or perceived stress more generally accounts for misophonia severity. A secondary aim was to conduct exploratory analyses to begin parsing apart mechanisms of trauma and stress-related sequalae (i.e., clusters of PTSD symptoms via the PCL-5) that contribute to misophonia severity.

Only 3.5% of participants met diagnostic criteria for current PTSD, with 21.7% meeting criteria for a lifetime diagnosis of PTSD. These results are generally consistent with previous studies using structured diagnostic interviews [6, 7, 24] and do not suggest a causative or unique association between misophonia and PTSD. The lifetime prevalence rate of PTSD within the current sample is, however, higher than the lifetime prevalence rate in a normative U.S. sample (estimated at 6.8–8%) [22, 45]. This discrepancy may in part be explained by the characteristics of our sample. Females are at higher risk for PTSD than males, and the majority of the current sample is female (74.4%), possibly contributing to higher lifetime prevalence. Another possible explanation for the higher prevalence of lifetime PTSD within the current sample could be that misophonia is associated with a lifetime history of PTSD, but not with a current diagnosis. This is speculative and would need to be replicated and explored more carefully, but it is plausible that people with misophonia may experience a diagnosis of PTSD at some point in their lifetime at a rate higher than the general population. Another hypothesis is that misophonia is a developmental risk factor for various psychiatric disorders, and not with PTSD specifically. Indeed, Rosenthal et al. reported that many lifetime psychiatric disorders (e.g., anxiety disorders, mood disorders) were observed at a higher rate than would be expected in a general population [7]. As such, it may be that PTSD is not unique in being one of many mental health problems higher in lifetime prevalence than in the general population.

Neither PTSD nor other trauma-related diagnoses accounted for misophonia in the current study. However, perceived stress explained a significant amount of the variance in misophonia severity, even after controlling for age, sex, and frequency of stressful life events across the lifespan. Misophonia is associated with factors known to be related to vulnerability to stress, including anxiety, neuroticism, and difficulties with emotion regulation [46–48]. One possibility is that the tendency to experience higher stress in general is associated with misophonia severity through these or other transdiagnostic factors. Another possible explanation for the relationship between higher misophonia and the tendency to generally experience greater stress (but not PTSD-related traumatic stress, per se) could be relatively simple: Individuals with misophonia symptoms experience higher stress because it is stressful to live with this disorder. There is no scientific evidence in the present study or elsewhere suggesting that traumatic stress or PTSD is a causal factor for misophonia. But perhaps misophonia is a causal factor for increased stress. Taking this hypothesis one step further, health problems associated with misophonia could be caused, in part, by transdiagnostic and treatable biological, social, and behavioral mechanistic factors underlying heightened stress. Future work exploring the components of stress and trauma as they relate to misophonia should also include dispositional factors and characterological features (e.g., neuroticism, difficulties with emotion regulation, *p* factor) in order to further our understanding of shared mechanisms.

It should be noted that in this study, we did not control for the temporality of stressful life events in relation to the onset of misophonia. As misophonia typically begins in childhood [2], it is important for future studies to examine the occurrence of childhood adverse events and the potential impact or overlap in development of misophonia. It is possible that there are shared transdiagnostic processes underlying both traumatic and misophonic etiology and sequelae. Just as unpleasant, inescapable, and uncontrollable situations are risk factors for PTSD, situations with misophonia triggers that are perceived as unpleasant, inescapable, and uncontrollable (i.e., in certain social or work settings, in childhood with limited autonomy) may exacerbate misophonia. Trauma and misophonia both lead to hyperarousal, efforts to avoid and/or escape, as well as pervasive attentional biases and interpersonal sensitivities. Taken together, there is clinically meaningful overlap in transdiagnostic factors between trauma and misophonia (e.g., perceived stress), but there is no evidence for a causal link between traumatic events and the development of misophonia.

In assessment and treatment of misophonia, then, it is important to understand idiographic *processes* related to stress more broadly (e.g., perceived stress), rather than simply probing trauma histories and related diagnoses. Further, our findings that PTSD symptomatology may be present even in the absence of a diagnosed trauma disorder imply that effective interventions for misophonia could involve a process-based approach with idiographic assessment and tailored interventions to target processes across biological, social, cognitive, and behavioral domains, rather than prescribing treatment that is specifically related to a history of traumatic stress. For example, if perceived stress is indeed a relevant mechanism for a patient, then interventions that are evidence-based and transdiagnostic for perceived stress could be considered as one part of a broader treatment strategy (e.g., implementing mindfulness-based stress reduction strategies that have been empirically tested for perceived stress [49, 50]. It will be valuable for future research to directly test evidence-based transdiagnostic interventions (i.e., stress reduction strategies for perceived stress) within misophonia samples to thoughtfully determine how to modify interventions for this population. For instance, mindfulness interventions largely have health benefits, but given sensitivities to sound in misophonia, the application of guided meditations, for example, need to be carefully modified for misophonia so that the mindfulness practices are not undermined by trigger sounds.

Considering processes of trauma sequalae that are often present in misophonia and not exclusively related to Criterion A events (e.g., hyperarousal) may be central to effective courses of treatment. Indeed, through analyzing individual symptom clusters of PTSD and their relationships to misophonia, this study also identified hyperarousal as a candidate transdiagnostic mechanism to assess and treat in misophonia populations. For example, if a patient presents for treatment of misophonia that has worsened in the context of a recent car accident, a first step may be to idiographically assess and develop a treatment plan given what that individual is willing and able to do in order to reduce hyperarousal that is maintaining heightened misophonic reactions. In collaboration with that patient, the provider may offer examples of process-based interventions that have been empirically supported to treat hyperarousal, such as applying progressive muscle relaxation before getting in a car with a family member or using emotion exposures from the Unified Protocol while driving or thinking about driving [51–53]. In line with the literature on effective process-based therapy, priority should be given to the intervention(s) that the patient is both able and willing to do, as well as to the intervention(s) that target processes of change that may be related to other relevant problems for that individual [54]. For example, a therapist and client may collaboratively decide to target hyperarousal first if functional analysis and ample assessment of the patient’s context suggests that decreasing hyperarousal before being triggered will then influence other associated processes (e.g., anger, irritability, aversive cognitions, anticipatory anxiety, avoidance behavior).

In any case, the complexity of interrelated constructs that contribute to misophonia severity underscore the importance of idiographic assessment and treatment development that thoughtfully apply either existing evidence-based treatments (e.g., the Unified Protocol) [55] or more tailored, idiographic interventions using a process-based approach [54]. Further, it is important in both assessment and treatment of misophonia to differentiate between PTSD and other trauma-related disorders, traumatic stress, and stress reactions more broadly. Interventions based on comprehensive assessment and that are designed to sequentially target features of a patient’s network across cognitive, affective, physiological, and attentional domains may work to target both misophonic and other stress or trauma-related suffering.

This study should be considered in the context of the limitations of the study. First, the sample was not representative of the U.S. population (https://www.census.gov/quickfacts/fact/table/US/PST045219), as the current sample had a higher percentage of White female participants, limiting generalizability of the results. Future research with more diverse and cross-cultural samples is needed to increase generalizability. Second, despite interviewing participants to gather both current and lifetime diagnoses, our data were cross-sectional, limiting definitive conclusions about causal relationships between relevant constructs over time. Future work that is longitudinal and prospective in nature will be crucial for bolstering our understanding of how misophonia, stress, and trauma interact, and for better accounting for the temporality of trauma exposure and onset of misophonia, increases in stress, and any trauma-related sequalae. Additionally, there is a need for more multi-trait multi-mode assessment of stress to better capture idiographic experience. Future work could incorporate behavioral tasks, as well as psychophysiology (e.g., galvanic skin response, heart rate), eye tracking, and facial behavior tracking to increase a granular understanding of mechanisms of stress and trauma (e.g., attentional biases, state-level distress). In addition, it is possible that participants in the current study misconstrued items on the PCL-5 self-report scale [56], and it is important to not conflate scores on the PCL-5 with a clinician-administered diagnosis of PTSD [57]. Results of the current study should be interpreted with this caveat in mind, and future work should incorporate clinician-rated tools to assess PTSD and trauma-related disorders more comprehensively. Despite these limitations, this study enhances our understanding that neither trauma history itself nor PTSD is directly related to misophonia, but transdiagnostic features of stress (e.g., perceived stress, hyperarousal) may contribute more strongly to misophonia severity.

The findings from the current study add to the nascent literature on the relationships among misophonia, stress, and trauma, and provide clinically meaningful implications for treatment of this understudied disorder. Prior studies have predominantly relied on self-report tools rather than rigorous clinical interviews to assess trauma diagnoses, and have not disentangled how varied elements of stress and trauma (e.g., perceived stress, cohort-level stressors, frequency of adverse life events, particular clusters of PTSD symptomatology) may differentially contribute to misophonia severity [6, 23, 24]. This study is the first to begin parsing apart mechanisms of stress broadly using both self-report and structured clinical assessment to assess how elements of perceived stress, stress during the ongoing COVID-19 pandemic, symptoms related to PTSD, and diagnosis of other trauma-related disorders (e.g., adjustment disorder, other specific trauma- and stressor-related disorder) are associated with misophonia. Further, we measured misophonia with the Duke Misophonia Questionnaire [38], a psychometrically validated tool that assesses misophonia holistically across trigger frequency, affective, physiological, cognitive and behavioral responses, as well as coping responses and impairment. The current study has implications for the development of more effective treatments for misophonia that take into account the complexities across stress, trauma, and misophonic reactions.
